# How to improve the well-being of patients in cardiac rehabilitation? A contribution of psychological treatments

**DOI:** 10.3389/fpsyg.2025.1592591

**Published:** 2025-06-13

**Authors:** Maura Crepaldi, Elisa Zambetti, Fiorella Lanfranchi, Emanuela Zenoni, Irene Bariletti, Francesco Quarenghi, Luigina Viscardi, Ginevra Rizzola, Valentina Regazzoni, Alessandra Bigoni, Francesca Brivio, Massimiliano Anselmi Kaiser, Irma Maria Soddu, Vittorio Giudici, Andrea Greco

**Affiliations:** ^1^Università degli Studi di Bergamo – Dipartimento di Scienze Umane e Sociali, Bergamo, Italy; ^2^Azienda Socio-Sanitaria Territoriale Bergamo Est – Struttura Semplice Dipartimentale di Psicologia, Seriate (Bergamo), Italy; ^3^Azienda Socio-Sanitaria Territoriale Bergamo Est – U. O. Specialistica di Riabilitazione Cardiovascolare, Seriate (Bergamo), Italy

**Keywords:** cardiovascular rehabilitation, psychological treatment, individual counseling, anxiety, depression, illness perception

## Abstract

**Introduction:**

The relationship between psychological factors and cardiovascular disease (CVD) has been extensively studied, with a focus on predictive factors and patients’ responses in the acute and chronic phases. However, less is known about the impact of specific psychological treatments on the psychological status of patients in cardiovascular rehabilitation.

**Methods:**

This study compares anxiety, depression and illness perception in patients with different CVD at three-time points: baseline (T0), end of the rehabilitation program (T1), and 3 months later (T2). One hundred and eighty-one patients in cardiovascular rehabilitation participated at three psychological treatments: psychoeducational intervention group, progressive muscle relaxation training with Jacobson’s technique and imaginative stabilization techniques, and individual counseling. The study included patients with Acute Coronary Syndrome (ACS), heart failure (HF), or undergoing cardiac surgery (CS), undergoing cardiovascular rehabilitation at ASST Bergamo Est between January and August 2023. Levels of anxiety, depression, and illness perception were measured by HADS and Brief-IPQ.

**Results:**

Significant differences were observed between T0, T1, and T2 in the three psychological treatments, with individual counseling showing the greatest improvements in anxiety, depression, and illness perception.

**Discussion:**

The study shows that individual counseling significantly reduces the levels of depression and anxiety, while promoting a healthier perception of the disease in comparison with the other two psychological treatments offered. This process is critical to improving the psychological well-being of patients, and these results could be useful to better address health care policies.

## 1 Introduction

Cardiovascular diseases (CVD) represent a significant challenge for global public health; along with cancer, chronic respiratory disease, and diabetes, they are among the most critical chronic noncommunicable diseases and rank as the leading cause of morbidity, disability and mortality, with a considerable impact at both the human and socioeconomic levels (World Health Organization, 1964). It stands as the leading cause of morbidity and mortality on a global scale (Türen et al., 2024).

The management of CVD has become more chronic and requires a comprehensive disease management approach that involves controlling behavioral risk factors and improving quality of life, both targeted through cardiac rehabilitation (Douma et al., 2024).

The medical community underestimates the role of psychological factors in adopting a healthy lifestyle among patients with CVDs (Greco et al., 2023).

Patient adherence to medical advice related to cardiovascular health is a crucial aspect of cardiac rehabilitation (CR). This involves sticking to cardiovascular risk-reducing behaviors such as regular physical activity, taking medication as prescribed, following a heart-healthy diet, managing stress, and quitting smoking (Lawton et al., 2022; Piepoli et al., 2010). Research has shown that adhering to these health behaviors can help prevent future cardiovascular events and mortality, underscoring the importance of patient adherence in cardiovascular health care (Douma et al., 2024).

Previous research has found practical and motivational barriers in CVD patients’ uptake of health behaviors, such as resistance to new lifestyles, time constraints, and lack of knowledge (Douma et al., 2024).

International guidelines, such as those of the European Society of Cardiology (ESC) and the American Heart Association (AHA), stress the comprehensive nature of a multidisciplinary approach. This approach, which combines the skills of physicians, dietitians, physical therapists, and psychologists, provides a robust strategy for achieving better prevention outcomes in all CVDs, from acute to decompensation (Byrne et al., 2024; Heidenreich et al., 2022; McDonagh et al., 2021; Piepoli et al., 2016).

CR is essential to improve well-being and promote health. The primary aim of CVD rehabilitation is to alleviate the physical and psychological burden and impact of the disease, enhance the quality of life for patients, prevent further cardiac events, effectively manage symptoms, and support patients’ return to an active lifestyle (Türen et al., 2024).

According to the World Health Organization (WHO), CR consists of a series of activities aimed at improving the mental, social, and physical well-being of individuals with heart disease. The goal is to help patients return to their normal professional and family life with their active participation (Kowalewska et al., 2024). It is divided into three phases: the first phase involves inpatient rehabilitation during hospitalization, the second phase includes physician-supervised physical activity after the patient’s discharge, and the third and final phase takes place on an outpatient basis. Patients must understand that following their physician’s guidance and adopting healthy behaviors are essential for preventing relapse (McMahon et al., 2017).

Recent studies have highlighted the significant impact of psychological risk factors, such as depression and anxiety, as well as protective factors like self-efficacy and adherence to therapy, on the course of CVD (Antonogeorgos et al., 2012; Blumenthal et al., 2012; Carbone et al., 2024; Franza et al., 2020; Tully et al., 2014).

In a meta-analysis conducted by Wrzeciono et al. (2024), it was found that therapies involving active patient participation lead to better outcomes. Specifically, CR combined with an intervention targeting psychological variables has the potential to be more effective in enhancing the quality of life for patients with CVD (Wrzeciono et al., 2024).

The application of psychological interventions in rehabilitation is a collaborative effort, with healthcare professionals playing a crucial role. Their efforts are aimed at reducing distress and psychological symptoms following the cardiovascular event, decreasing the impact of psychological risk factors on cardiovascular pathology, and promoting the modification of the patient’s problematic behaviors. This enhances the patient’s resources and the impact of his or her protective factors on the pathological prognosis, enabling the cardiopathic patient to recover from the cardiovascular event in a resilient manner (Fritzsche et al., 2020; Kupper et al., 2024).

Based on these premises, this study aims to test the effectiveness of psychological interventions in improving the mental health of patients undergoing cardiac rehabilitation program. Specifically, this study aims to compare the effect of three different types of psychological interventions: (a) psycho-educational intervention group, (b) progressive muscle relaxation training integrated with Jacobson’s technique and imaginative stabilization techniques, and (c) individual counseling on the levels of anxiety, depression and illness perception of patients suffering from CVDs at the beginning (T0), at the end (T1), and 3 months after a cardiac rehabilitation program (T2).

Starting from the literature presented (i.e., Türen et al., 2024; Wrzeciono et al., 2024), despite the nature of this study being exploratory, we hypothesize that patients who benefit from individualized treatment (treatments b and c) perceive better well-being than patients who do not.

## 2 Materials and methods

### 2.1 Design

The design of the present study is longitudinal, with three data collection points. Patients admitted to the cardiac rehabilitation unit were assigned, upon admission, to various combinations of psychological interventions (psychoeducational group, relaxation training, individual counseling): while participation to the psychoeducational group was mandatory for all participants, the admission criteria for relaxation training and individual counseling were determined by patients’ self-reported anxiety and depression levels, measured at the admission to the unit (T0). These levels were then reassessed at the end of the treatment, at the time of their discharge from the unit (T1) and 3 months later, during a follow-up conducted via telephone, once the patients had returned home (T2). Additionally, the different treatment groups were compared with one another regarding the progression of their respective levels of anxiety, depression, and illness perception across the three times of data collection. This approach allowed for the comparison of the effects of different types of psychological interventions on these psychological states and the evaluation of their efficacy.

The sample size was established *a priori* using G*Power 3.1 software (v.3.1.9.2, Heinrich-Heine-Universität Düsseldorf, Düsseldorf, Germany), the expected effect size was set at (Cohen’s f) 0.25, the α level was set at 0.05, and the power (1 − β) was set at 0.80, with the comparison of three groups and three measurements. The total sample size need to be almost 108.

### 2.2 Participants

The study involved 181 patients, 71.8% were men, the age range was 21–85 years (*M* = 65.96, *SD* = 11.90), and the average level of education was 10.27 years (*SD* = 3.79).

Participants were included based on the following inclusion criteria: diagnosis of CVD, enrollment in the unit’s multidisciplinary cardiac rehabilitation program, sufficient proficiency in the Italian language (since both the conducted cardiac rehabilitation program and the administered questionnaires were in Italian), and a minimum age of 18 years.

Specific clinical variables were evaluated, such as cardiac ejection fraction (EF) (normal, moderately reduced, severely reduced), body mass index (BMI) (underweight, normal weight, overweight, obesity) and the type of CVD (acute coronary syndrome (ACS), heart failure (HF), programmed cardiac surgery). Demographics of the final sample (181 participants) are reported in Table 1.

**Table 1:** Sociodemographic characteristic of participants.

	*N*	%
Gender		
Male	130	71.8
Female	51	28.2
Relationship status		
Married	108	71.1
Divorced	7	4.6
Single	7	4.6
Widowed	10	6.6
Other	20	13.2
Educational level (years)		
≤8 years	76	42
>8 years	105	58
EF		
≥55 (normal)	84	46.9
35–54 (moderately reduced)	56	31.3
<35 (severely reduced)	39	21.8
BMI		
<18 (underweight)	1	0.6
19–24 (normal weight)	88	49.7
25–29 (overweight)	62	35.0
≥30 (obesity)	26	14.7
Pathology		
Acute coronary syndrome (ACS)	36	19.9
Heart failure (HF)	25	13.8
Cardiac surgery (CS)	120	66.3

*N* = 181. Participants were on average 65.96 years old (*SD* = 11.90, range 21–85).

Among the 181 patients, 92.8% (*N* = 168) participated in the psychoeducational group, 66.3% (*N* = 120) attended relaxation training, and 19.3% (*N* = 35) received individual counseling sessions. Overall, 11.6% (*N* = 21) of patients engaged in all three types of psychological interventions. At T1 the number of total participants was 151 (with a participation rate of 83.43%), and 159 patients at T2 (with a participation rate of 87.85%). A total of 139 patients (76.80%) participated in all three stages.

### 2.3 Procedure

Data collection for the study was conducted from January 18, 2023, to November 27, 2023. Participant recruitment commenced upon the admission of patients to the Cardiac Rehabilitation Unit at ASST Bergamo Est. After obtaining informed consent participants were provided with detailed instructions on completing a self-administered questionnaire. This questionnaire included: the Italian version of the Hospital Anxiety and Depression Scale (HADS) (Costantini et al., 1999; Snaith, 2003) and the Italian version of the Brief Illness Perception Questionnaire (IPQ-B) (Ski et al., 2024). Participants then proceeded to complete the baseline questionnaire (T0).

Following this initial evaluation, patients commenced a multidisciplinary rehabilitation program that included pharmacological treatment for their cardiac condition, dietary education, physical exercise, and one or more of three psychological interventions. These interventions comprised participation in (a) a psychoeducational group, (b) engagement in relaxation training based on Jacobson’s progressive muscle relaxation and imaginative stabilization techniques, and (c) individual counseling. They were implemented within a stepped-care model, allowing patients to participate in one or more interventions.

The psychoeducational group intervention was administered to all patients except those with mobility or language impairments or those who declined participation in the study. Psycho-educational training consists of attending group meetings aimed at educating the patient to understand the disease more deeply in order to collaborate actively in treatment, empowering them and promoting adherence to treatment.

Relaxation training based on Jacobson’s progressive muscle relaxation and imaginative stabilization techniques was provided to patients exhibiting borderline or pathological levels of anxiety or depression, as indicated by a HADS’s score of 8 or higher. This intervention was also accessible upon patient request or cardiologist recommendation. Relaxation techniques represent a set of practices developed to reduce stress and promote psychophysical well-being by inducing the body’s natural relaxation response. These include progressive muscle relaxation, deep breathing, autogenic training, guided visualization and assisted biofeedback.

Individual counseling was administered to patients with pathological levels of anxiety or depression (HADS scores ≥ 11). Additionally, patients without pathological levels could request individual counseling based on cardiologist recommendation or personal preference; however, its availability was limited, due to the psychologists’ scheduling constraints. Individual counseling is a form of psychological intervention that provides patients emotional, informational, and behavioral support. Individual counseling is the psychological treatment that is best placed at the crossroads between interventions aimed at reducing psychological symptoms and those oriented at modifying risk behavior.

Each patient underwent a multidisciplinary rehabilitation program lasting approximately 2 weeks, after which a follow-up psychological assessment was conducted, typically on the day prior to discharge (T1). Three months post-discharge (T2), participants were re-contacted via telephone for a second follow-up assessment. At T2, patients did not complete self-report questionnaires; instead, they participated in a semi-structured telephone interview. During this interview, Hospital Anxiety and Depression Scale (HADS) and the Brief Illness Perception Questionnaire (IPQ-B), were administered.

### 2.4 Measures

Sociodemographic data were collected from the medical records of the recruited patients. The questionnaires administered remained consistent across the three data collection times (T0, T1, and T2).

The HADS (Snaith, 2003) is a self-report scale often administered in hospital settings to assess symptoms of anxiety and depression in patients. The Italian version of the HADS exhibits a high reliability (with Cronbach’s alpha coefficients ranging from 0.83 to 0.85 for both the anxiety (HADS-A) and depression (HADS-D) subscales) and directly measures symptoms of anxiety and depression reported by patients over the preceding 2 weeks. Similar to the original questionnaire, the Italian version comprises 14 items, with 7 items assessing anxiety symptoms and 7 items assessing depressive symptoms. Patients need to rate how much they agree with each item’s statement by selecting the most fitting answer out of 4 possible options. The options correspond to the points of a 4-point Likert scale ranging from 0 to 3, resulting in total scores ranging from 0 to 21 for both the anxiety and depression subscales. Scores are categorized as normal (0–7), borderline (8–10), or pathological (≥11), aiding in guiding subsequent clinical decisions. The scale showed an adequate internal consistency (Cronbach’s alpha was for HADS-A at T0 = 0.80, at T1 = 0.79, at T2 = 0.82; for HADS-D at T0 = 0.73, at T1 = 0.77 and at T2 = 0.81).

The Brief Illness Perception Questionnaire (IPQ-B) (Ski et al., 2024) comprises nine items evaluated on a Likert scale ranging from 1 (minimum) to 10 (maximum). The first five items assess patients’ cognitive perceptions of their illness, including impact on daily life, perceived duration, perceived controllability of the illness, beliefs about treatment efficacy, and symptom experience. Items 6 and 8 evaluate emotional aspects such as concern about the illness and perception of one’s emotional state. Item 7 measures the degree of illness understanding. The final item is open-ended, prompting participants to list the three most significant factors they believe are causing their illness. The version of the IPQ-B utilized in this study is based on the Italian validation (Ski et al., 2024) and excludes item 9 from evaluations. In the Italian validation, this item, which measured the cyclical timeline dimension (i.e., the belief that one’s illness symptoms tend to fluctuate and recur periodically over time, rather than being stable or linear), exhibited unacceptable internal reliability (Cronbach’s alpha = 0.41). Low illness perception scores indicate better levels of illness perception. The scale showed an adequate internal consistency (Cronbach’s alpha was at T0 = 0.72, at T1 = 0.80 and at T2 = 0.70).

### 2.5 Statistical analysis

Analyses were carried out using IBM SPSS Statistics software (29.0.1.0), to conduct general linear models (GLM) to test the trends of three psychological factors (depression, anxiety, and illness perception) at three detection times (T0, T1, and T2) for patients participating in one or more of three psychological interventions (psychoeducational group, relaxation training, individual counseling). To assess the impact of types of psychological treatment on changes in psychological factors between T0, T1, and T2, all relevant variables, such as sociodemographic and clinical factors (time, age, sex, cohabitation, educational level, BMI, FE, type of CVD, cardiac comorbidity, psychological comorbidity, and physical comorbidity) were included in the model to account for potential confounding effects.

## 3 Results

An analysis of the clinical characteristics of the patient sample revealed a mean Body Mass Index (BMI) of 25.50 (*SD* = 4.33), and a mean Ejection Fraction (EF) of 48.09% (*SD* = 12.80). Upon admission to the ward, patients were categorized into three clinical cardiac pathology groups, according to their diagnosis: acute coronary syndrome (ACS), heart failure (HF), and cardiac surgery. 120 (66.3%) were classified as cardiac surgery, 36 (19.9%) as ACS, and 25 (13.8%) as HF.

At baseline (T0), patients’ psychological status was evaluated. In terms of anxiety scores (*M* = 5.28; *SD* = 3.71), 76.8% (*N* = 139) demonstrated normal levels, 14.9% (*N* = 27) were borderline, and 8.3% (*N* = 15) were pathological. Regarding depression scores (*M* = 4.10; *SD* = 3.26), 84% (*N* = 152) of patients exhibited normal levels, 11% (*N* = 20) were classified as borderline, and 5% (*N* = 9) presented pathological levels. Illness perception reported a mean level of 4.96 (*SD* = 1.31).

At T1 patients’ level of anxiety scores (*M* = 4.10; *SD* = 3.06), 785.7% (*N* = 132) demonstrated normal levels, 11.0% (*N* = 17) were borderline, and 3.2% (*N* = 5) were pathological. Regarding depression scores (*M* = 2.92; *SD* = 2.90), 90.9% (*N* = 140) of patients exhibited normal levels, 5.8% (*N* = 9) were classified as borderline, and 3.2% (*N* = 5) presented pathological levels. Illness perception reported a mean level of 4.44 (*SD* = 1.32).

At T2 patients’ level of anxiety scores (*M* = 1.30; *SD* = 2.55), 96.9% (*N* = 154) demonstrated normal levels, 1.3% (*N* = 2) were borderline, and 1.9% (*N* = 3) were pathological. Regarding depression scores (*M* = 1.08; *SD* = 2.22), 97.5% (*N* = 155) of patients exhibited normal levels, 1.3% (*N* = 2) were classified as borderline, and 1.3% (*N* = 2) presented pathological levels. Illness perception reported a mean level of 2.79 (*SD* = 1.23).

The variables that predict a significant decrease in anxiety along the three times T0, T1, and T2 are time (*F*(2, 179) = 3.32, *p* = 0.040, *η*^2^ = 0.060) and cardiac pathology (*F*(2, 179) = 2.51, *p* = 0.043, *η*^2^ = 0.046); in addition, both psychological comorbidity (*F*(2, 179) = 5.02, *p* = 0.008, *η*^2^ *= 0*.088) and, among psychological treatments, individual counseling (*F*(2, 179) = 7.20, *p* = 0.001, *η*^2^ = 0.122), predict a highly significant decrease in anxiety along the three times.

**Figure 1:**
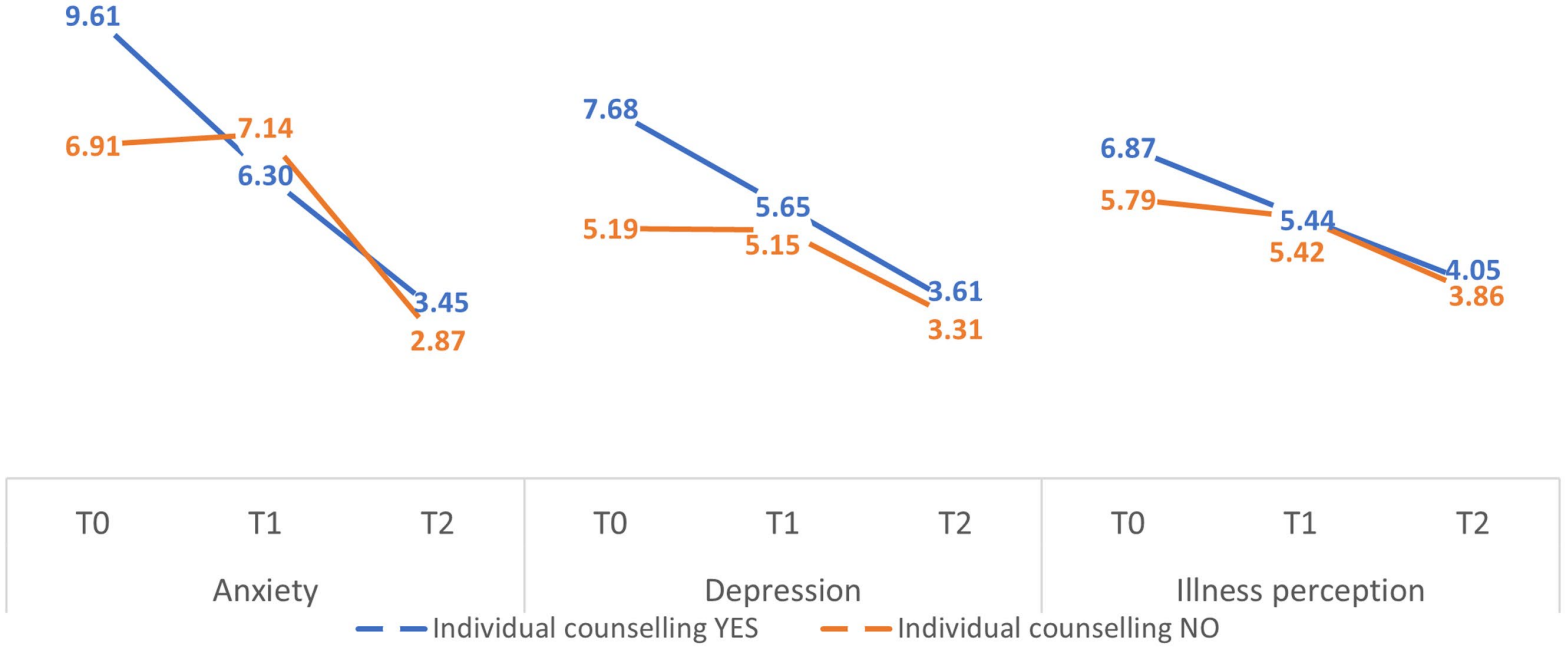
Trajectories in anxiety, depression, and illness perceptions levels.

Patients who participate in individual counseling at T0 show higher mean values of anxiety (Figure 1) (marginal mean (MM) = 9.61; standard error (SE) = 5.54) than the mean values of the sample (MM = 8.26; SE = 1.04) and the mean values of those who do not participate in individual counseling (MM = 6.91; SE = 1.14). At discharge from the ward (T1), at the end of treatment and rehabilitation in the hospital, patients who participate in individual counseling show lower mean values of anxiety (MM = 6.30; SE = 1.05) than both the sample mean values (MM = 6.72; SE = 0.89) and the mean values of those who do not participate in individual counseling (MM = 7.14; SE = 0.97). At T2, at the time of telephone follow-up, those who participated (MM = 3.45; SE = 0.81) in individual counseling show higher mean values of anxiety than the mean values of the sample (MM = 3.16; SE = 0.68) and of those who did not participate in individual counseling (MM = 2.87; SE = 0.75).

Regarding illness perception, individual counseling is the only variable that predicts a significant improvement (*F*(179) = 3.38, *p* = 0.038, *η*^2^ = 0.066). In particular, concerning the effect of individual counseling on improved levels of illness perception, patients participating in individual counseling leave (T0) with higher mean illness perception scores (MM = 6.87; SE = 0.51) than the mean scores of the sample (MM = 6.33; SE = 0.43) and the mean scores of those not participating in individual counseling (MM = 5.79; SE = 0.47). At discharge from the ward (T1), at the end of treatment and rehabilitation in hospital, patients who participate in individual counseling show a substantial decrease in the mean values of illness perception (MM = 5.44; SE = 0.54), which remain slightly higher than both the sample average (MM = 5.43; SE = 0.46) and the average of those who do not participate in individual counseling (MM = 5.42; SE = 0.51). Finally, at the time of telephone follow-up (T2), approximately four months after discharge (Mdays = 122.99; SDdays = 19.45), those who participated in individual counseling again showed a substantial decrease in the mean values for the illness perception (MM = 4.05; SE = 0.47), which remain slightly higher than the mean values for the sample (MM = 3.95; SE = 0.40) and for those who did not participate in individual counseling (MM = 3.86; SE = 0.44).

In particular, about the effect of individual counseling on the decrease in depression levels, patients participating in individual counseling leave (T0) with higher mean depression values (MM = 7.68; SE = 1.06) than the sample mean values (MM = 6.44; SE = 0.90) and the mean values of those not participating in individual counseling (MM = 5.19; SE = 0.94). On discharge from the ward (T1), at the end of treatment and rehabilitation in hospital, patients who participate in individual counseling show a substantial decrease in the mean values for depression (MM = 5.65; SE = 0.93), which remain higher than both the mean values for the sample (MM = 5.40; SE = 0.79) and the mean values for those who do not participate in individual counseling (MM = 5.15; SE = 0.86). Finally, at telephone follow-up (T2), about four months after discharge (Mdays = 122.99; SDdays = 19.45), those who took part in individual counseling again showed a substantial decrease in the mean values for depression (MM = 3.61; SE = 0.73), which remained higher than the mean values for the sample (MM = 3.46; SE = 0.62) and for those who did not take part in individual counseling (MM = 3.31; SE = 0.68).

## 4 Discussion

The aim of the study was to explore the effectiveness of three specific psychological interventions (psychoeducational intervention group, progressive muscle relaxation training integrated with Jacobson’s technique and imaginative stabilization techniques, and individual counseling) on reducing anxiety and depression and improving illness perception among patients undergoing a multidisciplinary cardiac rehabilitation program, by comparing these psychological variables at baseline (T0), end of the rehabilitation program (T1), and at a three-month follow-up (T2).

The literature on CVD shows that anxiety and depression are frequent in patients, with reported prevalence rates of around 40%, whether in individuals with coronary heart disease (CHD), heart failure (HF) or atrial fibrillation (AF). This symptomatology is associated with a reduced health-related quality of life (Pain et al., 2006).

Moreover, the results of this study partially confirm our hypothesis. The study show evidence for the efficacy of psychological interventions in improving the mental health of patients undergoing multidisciplinary cardiac rehabilitation programs; patients who benefit from individual counseling, perceive better well-being than patients who do not, also compared to progressive muscle relaxation training group. The results in terms of individual psychological treatment are in line with previous research (Wrzeciono et al., 2024; Albus et al., 2019) that reports the effectiveness of psychological treatments in cardiac rehabilitation, not only for reducing the psychological symptoms reported by patients but also for promoting and adopting healthy behaviors by reducing risky behaviors such as smoking, sedentary lifestyle, and inadequate diets. Suboptimal participation in cardiac rehabilitation is a well-documented issue that causes patients with CVD to miss opportunities to enhance their quality of life. Research has identified barriers and facilitators influencing patients’ commitment to cardiac rehabilitation and secondary prevention programs (Colivicchi et al., 2010). In line with the study presented, the literature shows that offering personalized and individualized care plans tailored to each patient’s needs is the most effective approach. Notably, our findings highlight the positive impact of individual counseling in decreasing the levels of depression and anxiety and contributing to the development of a more appropriate illness perception, thus playing an essential role in the psychophysical well-being of patients. Patients who participated in individual counseling sessions showed the greatest improvements in anxiety, depression and illness perception levels across the data collection points T0, T1, and T2. Despite presenting with higher anxiety, depression and maladaptive illness perception levels at baseline (T0), this intervention facilitated significant improvements by the end of the cardiac rehabilitation program (T1) and at the three-month follow-up (T2): by T1, patients that participated in individual counseling had quickly reached levels of anxiety, depression, and illness perception similar to those who had non-pathological levels at baseline, and maintained similar levels at the three-month follow up (T2).

The superior efficacy of individual counseling may be attributed to its personalized therapeutic approach. Unlike group interventions, individual counseling offers tailored strategies adapted to each patient’s unique emotional challenges and maladaptive cognitive beliefs following the traumatic experience of an acute CVD event. According to recent cardiac rehabilitation guidelines (Piepoli et al., 2016), psychological interventions aim both to modify the impact of psychosocial factors and risk behaviors - such as smoking, physical inactivity and maladaptive stress-coping strategies - and to assist individuals in emotionally adjusting to their illness. Among the psychological interventions provided in this study, individual counseling uniquely addresses both objectives by facilitating exploration and response to the patient’s specific needs through a therapeutic setting that allows for greater flexibility in addressing individual psychological processes. While psychoeducational group sessions help patients modify risk behaviors, increase awareness of maladaptive stress-coping strategies and improve stress management, and relaxation training targets symptoms of anxiety and depression to reduce their burden, individual counseling can effectively combine emotional adjustment to the illness - by mitigating the impact of emotional distress experienced following the acute CVD event - with the promotion of healthier behaviors, the development of more adaptive stress-coping strategies and improved therapeutic adherence through enhanced illness perception (Franza et al., 2020; Colivicchi et al., 2010; Galati and Vigorito, 2012; Niebauer, 2017; Tully et al., 2017; Coninx and Hansen, 2025). In contrast, while psychoeducational groups and relaxation training also contributed to improvements, their effects were less pronounced. These interventions provide valuable general information and stress management techniques but may not sufficiently address individual psychological complexities. The group setting may limit the opportunity for personalized feedback and exploration of individual concerns, which could explain the lesser impact compared to individual counseling.

These results underscore the complex interplay between psychological factors and CVDs, emphasizing the importance of a holistic approach to cardiac rehabilitation. Furthermore, the study highlights the necessity of multiple psychological interventions in multidisciplinary cardiac rehabilitation programs, incorporating individualized psychological interventions. Given the significant reductions in anxiety and depression and improvements in illness perception associated with individual counseling, healthcare providers should consider allocating resources to include personalized psychological support for cardiac patients. This approach could enhance patient adherence to medical advice, promote healthier lifestyles, and potentially reduce the risk of future cardiac events. Moreover, these results highlight the importance of early psychological assessment in CR settings. Identifying patients with elevated anxiety, depression, or maladaptive illness perceptions at admission allows for timely intervention, which can improve rehabilitation outcomes and overall patient well-being.

In conclusion, the data that emerged in the study align with literature evidence suggesting the importance of individualized patient care and that interdisciplinary health care programs that include diverse cardiovascular and mental health professionals and experts can effectively improve outcomes and reduce CVD-related costs (Coninx and Hansen, 2025).

In this perspective, the clinical benefits of interdisciplinary interventions are supported by research published in the literature, which guides the design of new, more functional treatment strategies and new effective trajectories that promote adherence to therapy, taking into account the multifaceted nature of these disorders.

Future studies should consider more extensive randomized controlled trials involving other inpatient rehabilitation facilities to validate these results and improve generalizability. In addition, investigating the long-term sustainability of psychological improvements with longer-term follow-ups would provide insights into the duration of the effects of individual counseling. Finally, exploring the specific components of individual counseling that contribute most to patient improvement could refine intervention strategies.

### 4.1 Limitations

This study has many strengths; however, it also has some limitations. The study employed a convenience sample from a single hospital unit, which may limit the generalizability of the findings. In the protocol there is no a control group since for ethical reasons psycho-educational treatment being offered as a matter of practice to all patients on the ward. However, the three types of treatment can be compared, considering the groups as internal controls. Additionally, attrition occurred at T1 and T2 due to patient unavailability or reluctance to continue participation, which could introduce bias. The reliance on self-reported measures may also be subject to response biases, such as social desirability or recall bias. A further limitation to highlight in the study is the change in the mode of data collection at T2. Although the same instruments (HADS and Brief-IPQ) were used at all three time points (T0, T1, and T2), the follow-up measurement at T2 was conducted by means of semi-structured telephone interviews, whereas at T0 and T1 self-administered questionnaires were used. Although this change in administration was useful in decreasing dropout, it is highlighted as a limitation of the study. In fact, T0 and T1 were carried out during the permanence in the hospital, instead T2 was conducted when patients were already at home.

A further limitation was the uneven distribution of males and females within the sample; in fact, males have a higher risk of developing CVD and therefore males are more prevalent in the cardiovascular population, future studies could involve more women.

## 5 Conclusion

The study’s results show that the type of psychological treatment during cardiac rehabilitation appears to affect patients’ psychological status, self-care, and disease management differently. Thus, it is essential to underscore the necessity of psychological interventions, particularly individual counseling, in cardiovascular rehabilitation settings. This is a crucial step in promoting patients’ health and well-being.

## Data Availability

The raw data supporting the conclusions of this article will be made available by the authors, without undue reservation.
